# Impact of image reconstruction on cerebral blood flow measured with ^15^O-water positron emission tomography

**DOI:** 10.1186/s40658-025-00760-5

**Published:** 2025-06-06

**Authors:** Elin Bäck, My Jonasson, Elin Lindström, Andreas Tolf, Joachim Burman, Lieuwe Appel, Mark Lubberink

**Affiliations:** 1 Molecular Imaging and Medical Physics, Department of Surgical Sciences, Uppsala University, Uppsala, Sweden; 2 Translational Neurology, Department of Medical Sciences, Uppsala University, Uppsala, Sweden

**Keywords:** ^15^O-water, Cerebral blood flow, Image reconstruction, Neuroimaging, Positron emission tomography

## Abstract

**Background:**

^15^O-water positron emission tomography (PET) is considered the gold standard method for non-invasive measurement of cerebral blood flow (CBF). However, previously published average CBF values in healthy subjects have varied greatly and the cause of these variations remains unclear. This study investigates how image reconstruction methods and spatial resolution affect CBF measurements with ^15^O-water PET.

**Methods:**

Eight healthy subjects each underwent dynamic ^15^O-water PET scans with continuous arterial blood sampling. Images were reconstructed using two different algorithms; ordered subset expectation maximisation and block sequential regularised expectation maximalisation with varying reconstruction parameters. CBF was estimated for the whole brain, grey matter, and central white matter. Reconstruction-specific effective spatial resolution was estimated using phantom measurements and simulations.

**Results:**

The mean whole brain CBF was 0.48 mL/cm^3^/min and showed little dependence on the image reconstruction method. Grey matter CBF varied between 0.52 and 0.57 mL/cm^3^/min, and central white matter CBF between 0.20 and 0.28 mL/cm^3^/min. Regional CBF showed great dependence on effective spatial resolution with a negative correlation between grey matter CBF and resolution (r = -0.96) and a positive correlation between central white matter and resolution (r = 0.93).

**Conclusion:**

This study concludes that grey matter and central white matter CBF, but not whole brain CBF measured with quantitative ^15^O-water PET is reconstruction method dependent, mainly due to varying spatial resolution with consequent partial volume effects. Variations in published CBF values cannot be explained solely by reconstruction methods or spatial resolution.

**Supplementary Information:**

The online version contains supplementary material available at 10.1186/s40658-025-00760-5.

## Background

In 1985, Lassen [1] stated that whole brain cerebral blood flow (CBF) in healthy adults aged 30–40 years is 50 mL/100 g/min and that reported values outside the range 45–55 mL/100 g/min must be caused by systematic methodological errors. He also noted that previously published CBF values, measured using various techniques, have ranged between 35 and 75 mL/100 g/min. Although methods have changed and been refined since then, for example with the introduction of non-invasive methods, the large variation in published CBF values persists. CBF is an important measurement in the diagnosis and monitoring of patients with a wide range of different neurological conditions including stroke, cerebrovascular disease, epilepsy, dementia, and head trauma [2]. Ensuring consistency of measured CBF values across methods and sites is imperative to future clinical application of CBF measurements.

CBF has been measured with ^15^O positron emission tomography (PET) for decades and the early methods, with either ^15^O-labelled carbon dioxide, oxygen gas or water, used steady-state and autoradiographic methods based on static PET scans [3, 4]. Today, dynamic ^15^O-water PET is considered the gold standard method for non-invasive CBF measurement [5]. ^15^O-water has a high extraction fraction which makes it an excellent perfusion tracer, as its uptake and clearance rates are closely related to tissue perfusion [6]. Absolute quantification of CBF still requires continuous arterial blood sampling to measure the patient's arterial blood activity throughout the scan. Although efforts have been made to negate the need for arterial cannulation, for example by using image-derived input functions [7, 8], these methods have yet to be fully validated for clinical use.

**Fig. 1:**
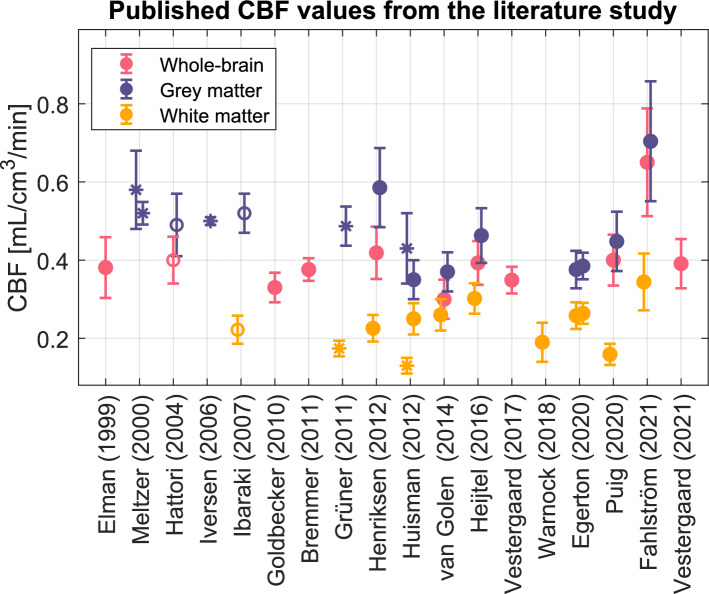
A selection of previously published mean CBF values with standard deviations in the whole brain, grey matter, and white matter. Filled circles indicate that the CBF values are derived from automatic segmentations, open circles from representative circular regions, and asterisks from manually drawn VOIs. Meltzer et al. [11] divided the study population into a younger and older group with mean ages of 29 and 70 years, respectively. Huisman et al. used two different segmentation methods on the same data [20]. Egerton et al. [39] is a test-retest study with CBF measured in the morning and afternoon. The Hattori et al. [14, 15] CBF values were published in two separate studies. The Fahlström et al. [18] data was recalculated using the same analysis method as the present study [7, 9–20, 36, 39–43]

In order to assess the variability in mean CBF estimates in studies using the dynamic bolus injection method, a small study of past and current literature was conducted for this introduction. The PubMed search term was: “^*15*^*O-water OR water OR H*_*2*_^*15*^*O OR *^*15*^*O) AND (PET OR “positron emission tomography”) AND (“cerebral blood flow” OR CBF) AND healthy*” with relevant journals included. Studies on healthy subjects at rest with fully quantitative methods, including bolus injections of ^15^O-water, dynamic acquisition, online arterial blood sampling, and kinetic modelling, were considered. In total, nineteen studies with unique data fulfilled the criteria and CBF values from these are found in Fig. 1. The mean whole brain CBF in healthy subjects varied between 0.30 and 0.65 mL/cm^3^/min, grey matter between 0.35 and 0.70 mL/cm^3^/min, and white matter between 0.13 and 0.34 mL/cm^3^/min.

This literature search showed that there is a large variation in methods between studies, such as the PET scanner used, bolus injection speed, acquisition and processing of the arterial input function, segmentation of volumes of interest (VOI), and reconstruction method, all of which could affect the final CBF value. The study populations consisted of either both sexes or only males and the mean age varied between 21 and 70 years. All studies except Grüner et al. [9], who used CT (computed tomography) images, used structural MRI (magnetic resonance imaging) images for co-registration and segmentation.

Reconstruction was either based on filtered back projection with a Hanning or Butterworth filter (all studies before 2010 [10–15], Bremmer et al. [16] and Warnock et al. [17], or iterative methods, typically ordered subset expectation maximisation (OSEM), which is the most used reconstruction method today. OSEM reconstruction was in one study [(Fahlström et al. [18])] done with the inclusion of time-of-flight (TOF), which improves convergence and spatial resolution, and in three studies (Grüner et al., Henriksen et al., Vestergaard et al. [7, 9, 19]) with point spread function (PSF) modelling. Gaussian post-filters with 2–6 mm kernels were applied for all OSEM reconstructions except Huisman et al. [20]. Studies have shown little difference between FBP and OSEM when it comes to quantification [21, 22]. More information on the studies can be found in the supplementary materials.

A recently implemented reconstruction method is the block sequential regularised expectation maximalisation (BSREM) algorithm (Q.Clear, GE Healthcare, Waukesha, WI) which penalises noise while iterating with the degree of penalisation indicated by the β-value. This method allows for full convergence without noise amplification, negating the need for smoothing filters.

Different reconstruction methods may result in different spatial resolutions, which are in turn expected to affect the final CBF value because of resulting partial volume effects. We hypothesise that this is an important contributor to the variations in reported CBF values in the literature. This study investigated the impact of various reconstruction methods and spatial resolution on quantitative CBF values measured with ^15^O-water PET in healthy subjects.

## Methods

### Subjects and data acquisition

Data from eight healthy subjects (5 females) with a median age of 41 (23–56) years were included from a completed research study at the Uppsala University Hospital. None of the included subjects had ongoing daily medication, they did not use tobacco and none had a history of cardiovascular, cerebrovascular, systemic inflammatory, hypertensive, or metabolic disease. Subjects fasted for two hours before the scan and abstained from caffeine and alcohol for at least twelve hours. Approval of the parent study was given by the local Radiation Ethics Committee and the Regional Medical Ethics Board in Uppsala (2014/453) and all subjects gave their written informed consent prior to participating.

Ten-minute dynamic ^15^O-water PET scans were performed on a Discovery MI PET/CT (GE Healthcare) consisting of 4 detector rings (20 cm axial field of view in 71 slices) [23]. A low-dose CT was acquired for attenuation correction. The PET scans started simultaneously with an automated controlled bolus injection of 5 MBq/kg ^15^O-water (5 mL at 1 mL/s followed by 35 mL saline at 2 mL/s). Online arterial blood sampling (at 3 mL/min) was performed during the entire scan (PBS-100, Veenstra-Comercer, Joure, The Netherlands). Discrete arterial blood samples were taken at 5 and 10 min post-injection and measured in a well-counter detector which had been cross-calibrated with the PET scanner. In addition, anatomical T1-weighted MRIs were acquired on an Achieva 3.0 T MRI scanner (Philips Healthcare, Best, The Netherlands), using a 32-channel head coil.

### Image reconstruction and motion correction

Emission data was reconstructed into 22 frames with durations 1 × 10, 8 × 5, 4 × 10, 2 × 15, 3 × 20, 2 × 30 and 2 × 60 s (6 min total) using a 128 × 128 × 71 matrix resulting in 1.97 × 1.97 × 2.79 mm^3^ voxels. Data was restricted to the first 6 min of the scan due to low counts resulting in noisy PET and blood sampling data at the end of the scan. All appropriate corrections were applied. Fourteen different OSEM reconstruction methods were used, with and without TOF and PSF and with varying numbers of iterations and subsets. A post-filter was applied to the OSEM reconstructions consisting of a variable transaxial 2D Gaussian kernel and a [1 4 1] weighted axial filter (‘standard z-filter’ in the Discovery MI software). Additionally, six BSREM reconstructions were used with varying degrees of noise penalisation (β-value). The reconstruction method recommended by the manufacturer, TOF-PSF-3i16s 5 mm, was chosen as the clinical reference method. The reconstruction methods are summarised in Table 1.

**Table 1:** Reconstruction parameters and post-filters

Reconstruction method	TOF	PSF	Iterations	Subsets	Gaussian filter [mm]	β-value
OSEM	No	No	3	16	3, 5, 7, 10	n/a
TOF-OSEM	Yes	No	3	16	5	n/a
TOF-PSF-OSEM	Yes	Yes	1, 2, 4, 5, 6	16	5	n/a
TOF-PSF-OSEM	Yes	Yes	3	16, 34	3, 5	n/a
BSREM	Yes	Yes	n/a	n/a	n/a	100, 200, 300, 400, 500, 600

OSEM: ordered subset expectation maximisation; TOF: time-of-flight; PSF: point spread function; BSREM: block sequential regularised expectation maximalisation

### Data processing and quantification

*Volumes of interest—*T1-weighted MRIs were co-registered to the PET images using rigid transformation and segmented into global grey and white matter masks using the PVElab probabilistic template with iterative thresholding [24]. The global grey matter mask was used as the grey matter (GM) VOI and a whole brain (WB) VOI was produced by adding the global grey and white matter masks. To minimise spill-over in white matter, a centrum semiovale white matter VOI (CWM) was generated by smoothing the global white matter mask with a 7 mm Gaussian filter and thresholding it adaptively to create a smaller 20 cm^3^ VOI [25]. Accordingly, WB, GM, and CWM time-activity curves were calculated by transferring the VOI masks to each frame of the dynamic PET images.

*Arterial input function—*Input functions were calibrated according to the discrete arterial blood samples taken during the scan. Dispersion of the arterial input function was estimated for each subject by iteratively fitting the single tissue compartment model with delay as a parameter and a range of dispersion times to the WB time-activity curve [26]. The individual optimal dispersion times and delays were determined by the lowest residual sum of squares regarding the fit of the model.

*Kinetic analysis*—CBF was quantified both at the regional and voxel level using the single tissue compartment model with fitted arterial blood volume ${V}_{A}$:

1 $$\begin{array}{*{20}c} {{\text{C}}_{{{\text{PET}}}} = \left( {1 - {\text{V}}_{{\text{A}}} } \right){\text{K}}_{1} {\text{C}}_{{\text{A}}} \left( {\text{t}} \right) \otimes e^{{ - k_{2} t}} + {\text{V}}_{A} {\text{C}}_{{\text{A}}} \left( {\text{t}} \right),} \\ \end{array}$$

with rate constants *K*_*1*_ and *k*_*2*_ for tracer uptake and clearance, respectively_,_ the input function *C*_*A*_ and time *t*, modelling the PET time-activity curve *C*_*PET*_. Equation (1) was fitted to the WB, GM, and CWM time-activity curves using non-linear regression to achieve regional estimates of *K*_*1*_ values. The convolution operation in the model applies an integration approach to interpolate the curve into the time frames of the PET scan. For the voxel-wise quantification method parametric *K*_*1*_ maps were produced using a basis function implementation of the single tissue compartment model [27, 28]. The mean *K*_*1*_ values in each of the WB, GM, and CWM VOIs were calculated.

The Shapiro–Wilk test for normality was performed on the CBF value of each reconstruction method and the Wilcoxon signed-rank test was used to test for significant differences between CBF values from each reconstruction method and the reference method. The median percentage differences between paired CBF values obtained by two different reconstruction methods were given.

### Effective spatial resolution measurement

To obtain the effective spatial resolution of each reconstruction method, phantom measurements were performed using the NEMA IQ phantom [29] on the Discovery MI PET/CT. The phantom consists of a 9.7 L tank with six fillable spheres with 10, 13, 17, 22, 28, and 37 mm internal diameters. The spheres and tank were filled with 22.3 and 2.2 kBq/mL ^68^Ga, respectively. ^68^Ga was used as a proxy for ^15^O because of their similar positron ranges, whereas the longer half-life of ^68^Ga allows for easier handling in a phantom measurement. The phantom was scanned for 7 min and image reconstruction was done using all methods in Table 1, but without applying any post-filters.

Simulated phantom images with spatial resolution between 1 and 14 mm full width at half maximum (FWHM) were created with 0.1 mm intervals by smoothing a mathematical representation of the NEMA IQ phantom with a 3D Gaussian filter. Recovery coefficient curves of the simulated phantom were calculated by dividing the mean activity in each sphere by the actual sphere activity, defining a relationship between each recovery curve and the simulated spatial resolution. Similarly, recovery coefficients were calculated for the measured phantom PET images. PET recovery curves were compared to the simulated recovery curves by residual sum of squares. The resolution of the simulated recovery curve with the smallest residual sum of squares was chosen as the effective spatial resolution of the PET image, disregarding post-filters. This calculation provides effective spatial resolution for BSREM reconstructions, since they are not filtered. For the OSEM reconstructions, the effective transaxial spatial resolution was calculated as the square root of the sum of the squared effective unfiltered spatial resolution and the squared post-filter size.

The relationship between effective resolution and CBF was analysed with linear regressions and the R^2^ value, a measure of how much the independent variable (resolution) explains the behaviour of the dependent variable (CBF). Pearson correlation coefficients and slope estimations were reported with a 95% confidence interval.

All data analysis and statistics were performed in MATLAB (R2022b) and figures were made using MATLAB (R2022b) and R (RStudio 2023.12.0).

## Results

**Fig. 2:**
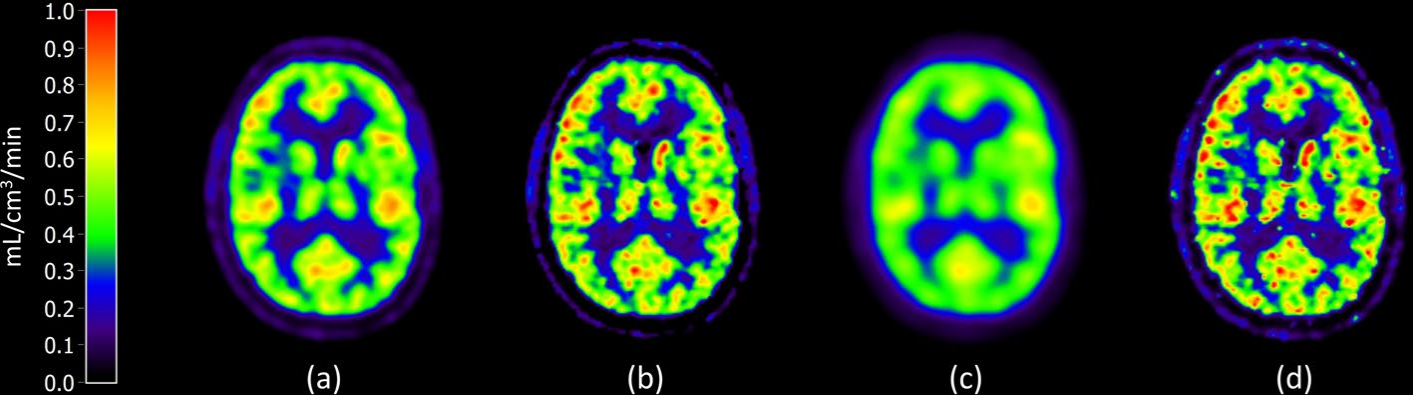
*K*_*1*_ maps of the same subject with different reconstruction methods. (**a**) TOF-PSF-3i16s 5 mm, the clinical reference method, (**b**) TOF-PSF-3i34s 3 mm, the sharpest OSEM reconstruction, (**c**) 3i16s 10 mm, lowest resolution reconstruction method, and (**d**) BSREM-β200, the second sharpest BSREM reconstruction

Large inter-subject variation in whole brain CBF was observed in this study with values ranging between 0.34 and 0.76 mL/cm^3^/min when using the reference reconstruction method TOF-PSF-3i16s 5 mm and voxel-wise quantification. GM CBF varied between 0.39 and 0.87 mL/cm^3^/min and CWM CBF between 0.15 and 0.29 mL/cm^3^/min, with the reference reconstruction. The normality of CBF values from each reconstruction method was confirmed with the Shapiro–Wilk test. A visual example of the difference between reconstruction methods can be found in Fig. 2 where parametric CBF maps from four different reconstruction methods are shown. A table of the distribution volume of water (${\text{K}}_{1}/{k}_{2}$ in Eq. (1)) for the regional quantification can be found in the supplementary materials. The arterial blood volume, ${V}_{A}$, was between 0% and 6% in WB, 0% and 7% in GM and 0% and 4% in CWM for the regional quantification.

Mean CBF values and standard deviations are given for each reconstruction method and VOI in Table 2, both for regional and voxel-wise quantifications. With regional quantification, the mean CBF values were 0.48–0.49 mL/cm^3^/min for WB, 0.52–0.57 mL/cm^3^/min for GM, and 0.20–0.28 mL/cm^3^/min for CWM. The mean CBF values were similar when using voxel-wise quantification, but with slightly larger variations. Between reconstruction methods, the mean CBF values were generally close to the reference (TOF-PSF-3i16s 5 mm) for the OSEM methods. BSREM reconstruction, however, resulted in slightly higher mean values in GM and slightly lower mean values in CWM. Reconstruction methods 3i16s 3 mm–10 mm deviate from the reference method with high CWM CBF values for both quantification methods. The GM/CWM ratios between mean CBF values ranged between 1.9:1 (3i16s 10 mm) and 2.9:1 (BSREM-β200) depending on reconstruction method.

**Table 2:** Mean CBF values and standard deviations for WB, GM, and CWM using regional and voxel-wise quantification

Reconstruction method	Whole brain (WB)		Grey matter (GM)		Centrum semiovale white matter (CWM)	
	Regional	Voxel-wise	Regional	Voxel-wise	Regional	Voxel-wise
3i16s 10 mm	0.48 ± 0.13	0.49 ± 0.12	0.52 ± 0.14	0.52 ± 0.13	0.28 ± 0.07	0.28 ± 0.06
3i16s 7 mm	0.48 ± 0.13*	0.49 ± 0.12*	0.53 ± 0.14	0.54 ± 0.13	0.26 ± 0.06	0.27 ± 0.06
3i16s 5 mm	0.49 ± 0.13	0.50 ± 0.12	0.54 ± 0.14	0.55 ± 0.14	0.25 ± 0.06	0.26 ± 0.06
3i16s 3 mm	0.49 ± 0.13	0.50 ± 0.13	0.55 ± 0.15*	0.56 ± 0.14	0.25 ± 0.06	0.26 ± 0.06
TOF-3i16s 5 mm	0.48 ± 0.13	0.49 ± 0.12	0.54 ± 0.14	0.55 ± 0.14	0.23 ± 0.05	0.23 ± 0.05
TOF-PSF-1i16s 5 mm	0.48 ± 0.13	0.49 ± 0.12	0.53 ± 0.14	0.54 ± 0.13	0.26 ± 0.06	0.26 ± 0.06
TOF-PSF-2i16s 5 mm	0.48 ± 0.13	0.49 ± 0.12	0.54 ± 0.15	0.55 ± 0.14	0.23 ± 0.05	0.23 ± 0.05
*TOF-PSF-3i16s 5 mm*	*0.48 ± 0.13*	*0.49 ± 0.12*	*0.55 ± 0.15*	*0.56 ± 0.14*	*0.22 ± 0.05*	*0.22 ± 0.05*
TOF-PSF-4i16s 5 mm	0.48 ± 0.13	0.50 ± 0.12	0.55 ± 0.15	0.56 ± 0.14	0.21 ± 0.05	0.22 ± 0.05
TOF-PSF-5i16s 5 mm	0.48 ± 0.13	0.50 ± 0.12	0.55 ± 0.15	0.56 ± 0.14	0.21 ± 0.05	0.22 ± 0.05
TOF-PSF-6i16s 5 mm	0.48 ± 0.13	0.50 ± 0.12	0.55 ± 0.15	0.56 ± 0.14	0.21 ± 0.05	0.22 ± 0.05
TOF-PSF-3i34s 5 mm	0.48 ± 0.13*	0.50 ± 0.12	0.55 ± 0.15	0.57 ± 0.14	0.21 ± 0.05	0.22 ± 0.05
TOF-PSF-3i16s 3 mm	0.48 ± 0.13	0.50 ± 0.12	0.56 ± 0.15	0.57 ± 0.14	0.21 ± 0.05	0.22 ± 0.05
TOF-PSF-3i34s 3 mm	0.49 ± 0.13	0.51 ± 0.13	0.56 ± 0.15	0.58 ± 0.15	0.21 ± 0.05	0.22 ± 0.05
BSREM-β100	0.49 ± 0.13	0.54 ± 0.13	0.57 ± 0.15	0.62 ± 0.15	0.20 ± 0.05	0.23 ± 0.05*
BSREM-β200	0.49 ± 0.13	0.52 ± 0.13	0.57 ± 0.15	0.60 ± 0.15	0.20 ± 0.05	0.21 ± 0.05
BSREM-β300	0.49 ± 0.13	0.51 ± 0.13	0.57 ± 0.15	0.59 ± 0.15	0.20 ± 0.05	0.21 ± 0.05
BSREM-β400	0.49 ± 0.13	0.51 ± 0.13	0.56 ± 0.15	0.58 ± 0.14	0.20 ± 0.05	0.22 ± 0.05
BSREM-β500	0.49 ± 0.13	0.51 ± 0.13	0.56 ± 0.15	0.58 ± 0.14	0.21 ± 0.05	0.22 ± 0.05
BSREM-β600	0.49 ± 0.13	0.50 ± 0.13	0.56 ± 0.15	0.57 ± 0.14	0.21 ± 0.05	0.22 ± 0.05

^*^Significant difference (p < 0.05 signed rank test) not reached with the reference reconstruction method TOF-PSF-3i16s 5 mm (italicised)

**Fig. 3:**
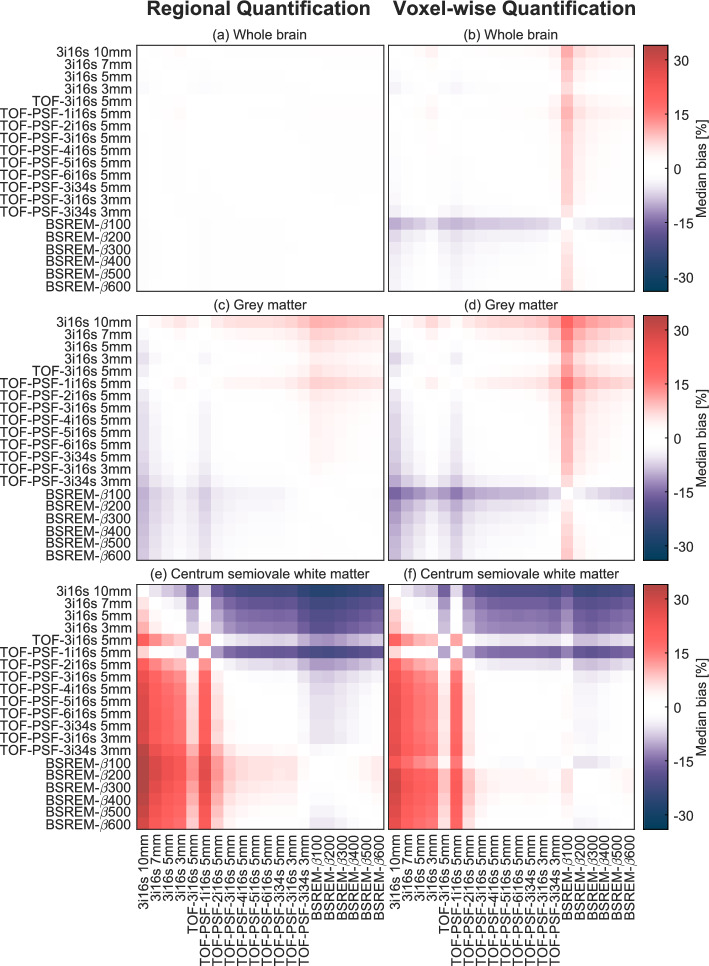
Heat maps showing median relative percentage difference between WB, GM, and CWM CBF values derived from images reconstructed with different methods for regional and voxel-wise quantification

Heat maps of the median relative percentage difference in WB, GM, and CWM CBF between all reconstruction methods and for both regional and voxel-wise quantification are shown in Fig. 3. For WB regional quantification, relative difference was consistently below 3%. For WB voxel-wise quantification, a relative difference below 7% was observed between all reconstruction methods except for BSREM-β100, see Fig. 3b. GM CBF varied more between reconstruction methods, with relative differences up to 10% for the regional method. GM differences for the voxel-wise quantification method were below 14% when excluding BSREM-β100 and up to 18% when including BSREM-β100, see Fig. 3d. For CWM CBF the relative differences display a group pattern, evident in Fig. 3e and f. With regional analysis, the difference in CWM CBF between reconstruction methods 3i16s 3 mm–10 mm was below 10%, and among the other methods (TOF-PSF-1i16s 5 mm excluded, see discussion) relative difference was below 13%. Comparing between these two groups, the maximum median difference was 36% and 30% for the regional and voxel-wise methods, respectively.

**Fig. 4:**
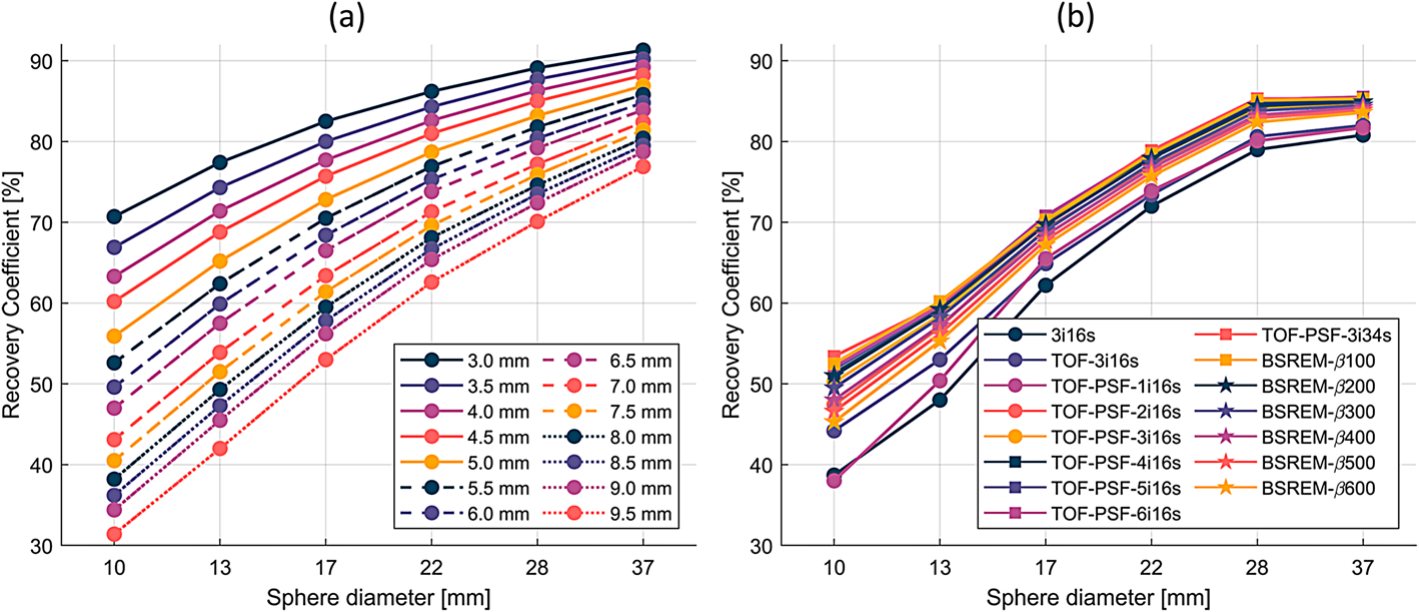
Recovery curves from the simulated NEMA phantom spheres (**a**) and the measured NEMA phantom PET images (**b**) reconstructed with the 15 methods in Table 1, without post-filter

Recovery curves of the simulated phantom, which the effective transaxial spatial resolution calculations are based on, and the recovery curves of the measured PET phantom images are found in Fig. 4. The effective transaxial spatial resolution of the OSEM reconstructions was found to be 8.2–12.6 mm for no-TOF, no-PSF (3i16s), with FWHM increasing with post-filter size. For TOF-3i16s 5 mm, it was 8.5 mm. TOF-PSF-(1–6)i16s 5 mm had an effective transaxial resolution of 7.4–8.8 mm, increasing FWHM with decreasing number of iterations. TOF-PSF-3i16s 3 mm had 6.5 mm, and TOF-PSF-3i34s 3 mm and 5 mm had 6.3 and 7.4 mm effective transaxial resolution, respectively. Effective resolution of the BSREM images was found to be between 5.5 and 6.6, with FHWM increasing with β-value.

**Fig. 5:**
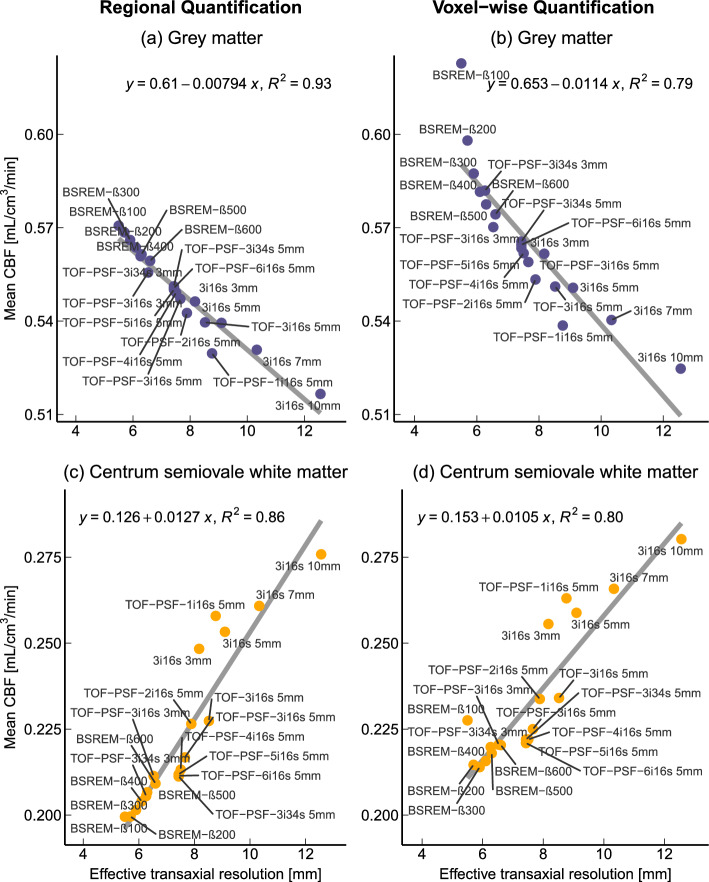
Mean GM and CWM CBF of each reconstruction method plotted over the effective transaxial reconstruction-specific resolution, with the linear regression line and R^2^ value

Figure 5 shows linear regressions between effective transaxial resolution and GM and CWM CBF. Figure 5a and b shows a strong negative relationship between mean GM CBF and effective transaxial resolution with correlation coefficients -0.96 [-0.99 to -0.91] and -0.89 [-0.96 to -0.73] and slopes -0.0079 [-0.0090 to -0.0069] and -0.0114 [-0.0144 to -0.0085] for the regional and voxel-wise methods, respectively. The opposite relationship is seen between CWM CBF and resolution in Fig. 5c and d, with correlation coefficients 0.93 [0.83 0.97] and 0.89 [0.74 0.96] and slopes 0.0127 [0.0102 0.0152] and 0.0105 [0.0079 0.0131] for the regional and voxel-wise methods, respectively. In GM and CWM, the effective transaxial resolution explains most of the variation in mean CBF, between 79% and 93%, indicated by the R^2^ values in Fig. 5. The correlation between mean WB CBF and effective resolution was -0.65 [-0.85 to -0.29] and -0.73 [-0.89 to -0.42] for regional and voxel-wise quantification, respectively, and the slope of the linear regression was -0.0014 [-0.0022 to -0.0006] and -0.0052 [-0.0076 to -0.0028] for the regional and voxel-wise methods, respectively. A figure of the linear regressions for the WB region can be found in the supplementary materials.

## Discussion

In this study, we investigated how image reconstruction methods and spatial resolution affect CBF measurements with ^15^O-water PET. Using a wide range of settings and post-filters to reconstruct the same eight scans of healthy subjects we showed that the effect of spatial resolution on whole brain CBF was negligible. However, the choice of reconstruction method and post-filter had a significant impact on GM and CWM CBF, for both the regional or voxel-wise quantification methods.

There were significant differences between CBF values of different reconstruction methods in all but five cases, see Table 2. This does not necessarily indicate a meaningful difference, only that a change in reconstruction method typically changes each individual’s CBF value in the same direction.

Figure 5 shows strong linear relationships between resolution and GM and CWM CBF, along with high R^2^ values, indicating that resolution can explain most of the variation in GM and CWM CBF. This suggests that partial volume effects are the main cause of the differences in CBF values derived from images reconstructed using different reconstruction methods. This hypothesis is further supported by the negligible median difference in WB CBF and the large differences in GM and CWM CBF as shown in Fig. 3.

As seen in Fig. 5, the mean CBF follows the regression line for most reconstruction methods, with two notable exceptions. Firstly, images reconstructed with TOF-PSF and a single OSEM iteration have lower GM and higher CWM CBF than predicted by the regression, likely because the reconstruction algorithm fails to converge. Secondly, CBF values from BSREM-β100, which is the sharpest but also assumed to be the noisiest reconstruction due to the low level of regularisation, behave differently depending on the quantification method. BSREM-β100 CBF values deviate greatly from the regression line in Fig. 5b and d, but not in Fig. 5a and c. A likely explanation is that when averaging is not done until after kinetic modelling, as with voxel-wise quantification, the noise propagates in the basis function algorithm with a bias towards high voxel values which skews the mean voxel-wise CBF.

In Hsu et al. [23], the transaxial spatial resolution of the Discovery MI PET/CT (the same scanner used in the present study) was determined following the NEMA NU-2 2012 standard. They found that the transaxial spatial resolution was 3.67 to 4.00 mm at 1 cm offset, 3.82 to 4.76 mm at 10 cm offset, and 4.31 to 7.44 mm at 20 cm offset when using OSEM-PSF reconstruction. The NEMA NU-2 standard defines the spatial resolution as the FWHM of capillary tubes filled with ^18^F. In the present study, spatial resolution was based on the amount of spill-out from hot spheres, which is more similar to a clinical setting compared to the NEMA NU-2 standard. With this method, the FWHMs were generally larger than those of Hsu et al., especially for the OSEM reconstructions. A resolution based on a clinically similar geometry is expected to be more accurate in the context of a partial volume correction. The point/line source model for calculating resolution has little clinical relevance and underestimates the effective resolution of clinical images. Use of a brain-specific phantom, for example, the Hoffman phantom [30], might have resulted in a somewhat more accurate estimation of partial volume effects, but would not have changed the general conclusion of this study.

With any of the reconstruction methods examined, mean CBF values in all three regions fall within the range of previously published values, see Table 2 and Fig. 1. Additionally, the mean WB CBF values are well within the range of 45–55 mL/100 g/min, given by Lassen [1]. Since the results of the present study could not explain the entire range of published CBF values, the question then stands on what has caused these variations. Some possible factors are discussed below.

The reliability of CBF measured with ^15^O-water PET greatly depends on the validity of the arterial input functions because errors in the amplitude of the input function propagate directly into errors in K_1_. There is no universally used standardised protocol for obtaining the input function and there are many possible sources of variations and error in the measuring and processing of it. These include sampling frequency, detector instability, calibration, and variations in tube length and diameter. Additionally, uncertainties in the delay and dispersion corrections also contribute to errors in the resulting CBF values.

The lack of standardised VOI makes comparing different studies challenging. The study by Huisman et al. [20] illustrates that variations in VOI definition greatly influence resulting CBF values. The manually drawn grey matter VOI was the insular grey matter and the manual white matter VOI was the centrum semiovale, each in four and two successive transversal planes, respectively. The automatic segmentation generated total grey matter and global white matter VOIs. These give widely different CBF values for grey and white matter, as seen in Fig. 1.

It is also worth noting that VOI definition methods also affect the grey-to-white matter CBF ratio. In the Huisman et al. [20] study, manually defined VOIs yielded a ratio of 3.3:1 while the automatic VOIs yielded a 1.4:1 ratio. All other studies included in Fig. 1 that have presented both grey and white matter CBF, had ratios within this range, with automatic segmentations generally giving lower ratios. In the present study it was also evident that the choice of reconstruction method greatly affects the grey-to-white matter ratio with the lowest ratio 1.9:1 from the lowest resolution reconstruction method, 3i16s 10 mm, and the highest ratio of 2.9:1 from the second to highest resolution reconstruction method, BSREM-β200. Since VOI and resolution affect the ratio, this is likely caused by partial volume effects.

Factors known to affect CBF in healthy individuals include sex, age, and caffeine intake. Sex and age have minor but significant effects on PET-measured CBF [31, 32]. However, Meltzer et al. [11] stated that the significant difference in mean CBF between the two age groups seen in Fig. 1 is resolved after applying a partial volume correction. Studies may, or may not, control for caffeine which is a known vasoconstrictor that lowers CBF [33]. In the present work, both females and males were included and there was a wide range in age (23–56), which could help explain the relatively wide range in individual CBF mentioned in the results.

As in the present study, sample sizes in healthy control PET studies are typically small, usually with less than 20 subjects, and conclusions on a population level should be avoided. It is worth mentioning that attempts have been made to create inter-centre normal CBF databases where larger CBF datasets would be available. A notable example is Ito et al. [34] where a total of 70 subjects across eleven centres in Japan were included with the conclusion that inter-centre variation in CBF was insignificant. This study included several different ^15^O-labelled tracers and methods, including ^15^O-CO_2_ inhalation.

In the introduction of this study, both PET/CT and PET/MRI studies were referenced, but no clear distinction in CBF between anatomical imaging modalities could be established. It is possible that the acoustic MRI noise may affect the research subjects and could cause heightened activity of the brain [35] and thus increase CBF values. To assess any analysis methodology influences in the Fahlström et al. [18] PET/MRI study, we re-analysed the data with the same analysis method used in the present work, resulting in similarly unusually high GM CBF values as published. However, the other PET/MRI studies by Puig et al. [36] and Vestergaard et al. [7] acquired on a different PET/MRI system, both show more typical CBF values.

Recent developments towards commercially available point-of-care chemistry and administration systems for ^15^O-water and an ongoing phase-3 study aiming for approval of its clinical use in the diagnosis of coronary artery disease in the US [37] may also result in more widespread availability of ^15^O-water for clinical and research investigations of CBF. For ^15^O-water PET to be a clinically viable method for measuring CBF, the issue with partial volume effects must be addressed. Widespread guidelines, such as the EANM’s EARL standard for FDG brain [38], could be utilised to harmonise the CBF imaging procedure. Such a standard should include a robust partial volume correction based on the effective resolution of the scanner and reconstruction method used. One could also opt to use the efflux of tracer from tissue, *k*_*2*_*,* to measure CBF, with the added complexity of requiring the distribution volume of water in brain tissues. The CBF values would be very sensitive to the assumed and fixed distribution volumes for each region, but the advantage of using *k*_*2*_ over *K*_*1*_ as a basis for CBF is that *k*_*2*_ is insensitive to errors in the calibration of the input function and less sensitive to partial volume effects.

## Conclusion

Whole brain CBF measured with ^15^O-water PET is independent of the reconstruction method. Grey and central white matter CBF, on the other hand, are highly dependent on the reconstruction method mostly due to varying degrees of partial volume effects caused by the different spatial resolutions of reconstruction methods. Regional versus voxel-wise quantification had only a minor impact on the CBF estimates. Reconstruction methods alone cannot explain the large variations observed in published CBF values.

## Supplementary Information

Supplementary Material 1.

Supplementary Material 2.

Supplementary Material 3.

## Data Availability

The datasets analysed during the current study are available from the corresponding author on reasonable request and with appropriate ethics permits.

## Abbreviations

1TCM: Single tissue compartment model
BSREM: Block sequential regularised expectation maximalisation
CBF: Cerebral blood flow
CT: Computed tomography
CWM: Centrum semiovale white matter
FWHM: Full width at half maximum
GM: Grey matter
MRI: Magnetic resonance imaging
OSEM: Ordered subset expectation maximisation
PET: Positron emission tomography
PSF: Point spread function (modelling)
TOF: Time-of-flight
VOI: Volume of interest
WB: Whole brain

## References

[1] Lassen NA. Normal average value of cerebral blood flow in younger adults is 50 ml/100 g/min. J Cereb Blood Flow Metab. 1985;5(3):347–9. 4030914 10.1038/jcbfm.1985.48

[2] Kaechele AP, Chakko MN. Nuclear medicine cerebral perfusion scan. Treasure Island (FL): StatPearls Publishing; 2023. 35881740

[3] Frackowiak RS, Lenzi GL, Jones T, Heather JD. Quantitative measurement of regional cerebral blood flow and oxygen metabolism in man using 15O and positron emission tomography: theory, procedure, and normal values. J Comput Assist Tomogr. 1980;4(6):727–36. 6971299 10.1097/00004728-198012000-00001

[4] Herscovitch P, Markham J, Raichle ME. Brain blood flow measured with intravenous H2(15)O. I. Theory and error analysis. J Nucl Med Off Publ Soc Nucl Med. 1983;24(9):782–9. 6604139

[5] Slart RHJA, Martinez-Lucio TS, Boersma HH, Borra RH, Cornelissen B, Dierckx RAJO, et al. [15O]H2O PET: potential or essential for molecular imaging? Semin Nucl Med. 2024;54(5):761–73. 37640631 10.1053/j.semnuclmed.2023.08.002

[6] Herscovitch P, Raichle ME, Kilbourn MR, Welch MJ. Positron emission tomographic measurement of cerebral blood flow and permeability-surface area product of water using [15O]water and [11C]butanol. J Cereb Blood Flow Metab Off J Int Soc Cereb Blood Flow Metab. 1987;7(5):527–42. 10.1038/jcbfm.1987.102 3498732

[7] Vestergaard MB, Calvo OP, Hansen AE, Rosenbaum S, Larsson HBW, Henriksen OM, et al. Validation of kinetic modeling of [15O]H2O PET using an image derived input function on hybrid PET/MRI. Neuroimage. 2021;233: 117950. 33716159 10.1016/j.neuroimage.2021.117950

[8] Young P, Appel L, Tolf A, Kosmidis S, Burman J, Rieckmann A, et al. Image-derived input functions from dynamic 15O–water PET scans using penalised reconstruction. EJNMMI Phys. 2023;10(1):15. 36881266 10.1186/s40658-023-00535-w PMC9992469

[9] Grüner JM, Paamand R, Højgaard L, Law I. Brain perfusion CT compared with15O-H2O-PET in healthy subjects. EJNMMI Res. 2011;1(1):28. 22214473 10.1186/2191-219X-1-28 PMC3251173

[10] Iversen P, Hansen DA, Bender D, Rodell A, Munk OL, Cumming P, et al. Peripheral benzodiazepine receptors in the brain of cirrhosis patients with manifest hepatic encephalopathy. Eur J Nucl Med Mol Imaging. 2006;33(7):810–6. 16550382 10.1007/s00259-005-0052-8

[11] Meltzer CC, Cantwell MN, Greer PJ, Ben-Eliezer D, Smith G, Frank G, et al. Does cerebral blood flow decline in healthy aging? A pet study with partial-volume correction. J Nucl Med. 2000;41(11):1842–8. 11079492

[12] Elman I, Sokoloff L, Adler CM, Weisenfeld N, Breier A. The effects of pharmacological doses of 2-deoxyglucose on cerebral blood flow in healthy volunteers. Brain Res. 1999;815(2):243–9. 9878763 10.1016/s0006-8993(98)01137-8

[13] Ibaraki M, Ito H, Shimosegawa E, Toyoshima H, Ishigame K, Takahashi K, et al. Cerebral vascular mean transit time in healthy humans: a comparative study with PET and dynamic susceptibility contrast-enhanced MRI. J Cereb Blood Flow Metab Off J Int Soc Cereb Blood Flow Metab. 2007;27(2):404–13. 10.1038/sj.jcbfm.9600337 16736045

[14] Hattori N, Bergsneider M, Wu HM, Glenn TC, Vespa PM, Hovda DA, et al. Accuracy of a method using short inhalation of (15)O-O(2) for measuring cerebral oxygen extraction fraction with PET in healthy humans. J Nucl Med Off Publ Soc Nucl Med. 2004;45(5):765–70. 15136624

[15] Hattori N, Huang SC, Wu HM, Liao W, Glenn TC, Vespa PM, et al. Acute changes in regional cerebral (18)F-FDG kinetics in patients with traumatic brain injury. J Nucl Med Off Publ Soc Nucl Med. 2004;45(5):775–83. 15136626

[16] Bremmer JP, van Berckel BNM, Persoon S, Kappelle LJ, Lammertsma AA, Kloet R, et al. Day-to-day test-retest variability of CBF, CMRO2, and OEF measurements using dynamic 15O PET studies. Mol Imaging Biol. 2011;13(4):759–68. 20700768 10.1007/s11307-010-0382-1 PMC3128261

[17] Warnock G, Sommerauer M, Mu L, Pla Gonzalez G, Geistlich S, Treyer V, et al. A first-in-man PET study of [18F]PSS232, a fluorinated ABP688 derivative for imaging metabotropic glutamate receptor subtype 5. Eur J Nucl Med Mol Imaging. 2018;45(6):1041–51. 29177707 10.1007/s00259-017-3879-x

[18] Fahlström M, Appel L, Kumlien E, Danfors T, Engström M, Wikström J, et al. Evaluation of arterial spin labeling MRI—comparison with 15O-water PET on an integrated PET/MR scanner. Diagnostics. 2021;11(5):821. 34062847 10.3390/diagnostics11050821 PMC8147295

[19] Henriksen OM, Larsson HBW, Hansen AE, Grüner JM, Law I, Rostrup E. Estimation of intersubject variability of cerebral blood flow measurements using MRI and positron emission tomography. J Magn Reson Imaging JMRI. 2012;35(6):1290–9. 22246715 10.1002/jmri.23579

[20] Huisman MC, van Golen LW, Hoetjes NJ, Greuter HN, Schober P, Ijzerman RG, et al. Cerebral blood flow and glucose metabolism in healthy volunteers measured using a high-resolution PET scanner. EJNMMI Res. 2012;2(1):63. 23168248 10.1186/2191-219X-2-63 PMC3544653

[21] Boellaard R, van Lingen A, Lammertsma AA. Experimental and clinical evaluation of iterative reconstruction (OSEM) in dynamic PET: quantitative characteristics and effects on kinetic modeling. J Nucl Med. 2001;42(5):808–17. 11337581

[22] Oda K, Toyama H, Uemura K, Ikoma Y, Kimura Y, Senda M. Comparison of parametric FBP and OS-EM reconstruction algorithm images for PET dynamic study. Ann Nucl Med. 2001;15(5):417–23. 11758946 10.1007/BF02988345

[23] Hsu DFC, Ilan E, Peterson WT, Uribe J, Lubberink M, Levin CS. Studies of a next-generation silicon-photomultiplier–based time-of-flight PET/CT system. J Nucl Med. 2017;58(9):1511–8. 28450566 10.2967/jnumed.117.189514

[24] Svarer C, Madsen K, Hasselbalch SG, Pinborg LH, Haugbøl S, Frøkjaer VG, et al. MR-based automatic delineation of volumes of interest in human brain PET images using probability maps. Neuroimage. 2005;24(4):969–79. 15670674 10.1016/j.neuroimage.2004.10.017

[25] Koole M, van Aalst J, Devrome M, Mertens N, Serdons K, Lacroix B, et al. Quantifying SV2A density and drug occupancy in the human brain using [11C]UCB-J PET imaging and subcortical white matter as reference tissue. Eur J Nucl Med Mol Imaging. 2019;46(2):396–406. 30121895 10.1007/s00259-018-4119-8

[26] Meyer E. Simultaneous correction for tracer arrival delay and dispersion in CBF measurements by the H215O autoradiographic method and dynamic PET. J Nucl Med. 1989;30(6):1069–78. 2786948

[27] Watabe H, Jino H, Kawachi N, Teramoto N, Hayashi T, Ohta Y, et al. Parametric imaging of myocardial blood flow with 15O-water and PET using the basis function method. J Nucl Med. 2005;46(7):1219–24. 16000292

[28] Boellaard R, Knaapen P, Rijbroek A, Luurtsema GJJ, Lammertsma AA. Evaluation of basis function and linear least squares methods for generating parametric blood flow images using 15O-water and positron emission tomography. Mol Imaging Biol. 2005;7(4):273–85. 16080023 10.1007/s11307-005-0007-2

[29] Caribé PRRV, Koole M, D’Asseler Y, Deller TW, Van Laere K, Vandenberghe S. NEMA NU 2–2007 performance characteristics of GE Signa integrated PET/MR for different PET isotopes. EJNMMI Phys. 2019;4(6):11. 10.1186/s40658-019-0247-x PMC6609673 31273558

[30] Harrison RL, Elston BF, Byrd DW, Alessio AM, Swanson KR, Kinahan PE. Technical Note: a digital reference object representing Hoffman’s 3D brain phantom for PET scanner simulations. Med Phys. 2020;47(3):1174–80. 31913507 10.1002/mp.14012

[31] Leenders KL, Perani D, Lammertsma AA, Heather JD, Buckingham P, Healy MJ, et al. Cerebral blood flow, blood volume and oxygen utilization: normal values and effect of age. Brain J Neurol. 1990;113(Pt 1):27–47. 10.1093/brain/113.1.27 2302536

[32] Esposito G, Van Horn JD, Weinberger DR, Berman KF. Gender differences in cerebral blood flow as a function of cognitive state with PET. J Nucl Med Off Publ Soc Nucl Med. 1996;37(4):559–64. 8691239

[33] Addicott MA, Yang LL, Peiffer AM, Burnett LR, Burdette JH, Chen MY, et al. The effect of daily caffeine use on cerebral blood flow: how much caffeine can we tolerate? Hum Brain Mapp. 2009;30(10):3102–14. 19219847 10.1002/hbm.20732 PMC2748160

[34] Ito H, Kanno I, Kato C, Sasaki T, Ishii K, Ouchi Y, et al. Database of normal human cerebral blood flow, cerebral blood volume, cerebral oxygen extraction fraction and cerebral metabolic rate of oxygen measured by positron emission tomography with 15O-labelled carbon dioxide or water, carbon monoxide and oxygen: a multicentre study in Japan. Eur J Nucl Med Mol Imaging. 2004;31(5):635–43. 14730405 10.1007/s00259-003-1430-8

[35] Sousa JM, Appel L, Engström M, Nyholm D, Ahlström H, Lubberink M. Comparison of quantitative [11C]PE2I brain PET studies between an integrated PET/MR and a stand-alone PET system. Phys Med. 2024;1(117): 103185. 10.1016/j.ejmp.2023.103185 38042064

[36] Puig O, Henriksen OM, Vestergaard MB, Hansen AE, Andersen FL, Ladefoged CN, et al. Comparison of simultaneous arterial spin labeling MRI and 15O–H2O PET measurements of regional cerebral blood flow in rest and altered perfusion states. J Cereb Blood Flow Metab Off J Int Soc Cereb Blood Flow Metab. 2020;40(8):1621–33. 10.1177/0271678X19874643 PMC7370368 31500521

[37] Di Carli MF, Gormsen LC, Chareonthaitawee P, Johnson GB, Beanlands R, DeKemp R, et al. Rationale and design of the RAPID-WATER-FLOW trial: radiolabeled perfusion to identify coronary artery disease using water to evaluate responses of myocardial FLOW. J Nucl Cardiol Off Publ Am Soc Nucl Cardiol. 2024;31: 101779. 10.1016/j.nuclcard.2023.101779 38215598

[38] Guedj E, Varrone A, Boellaard R, Albert NL, Barthel H, van Berckel B, et al. EANM procedure guidelines for brain PET imaging using [18F]FDG, version 3. Eur J Nucl Med Mol Imaging. 2022;49(2):632. 34882261 10.1007/s00259-021-05603-w PMC8803744

[39] Egerton A, Dunn JT, Singh N, Yu Z, O’Doherty J, Koychev I, et al. Evaluation of [13N]ammonia positron emission tomography as a potential method for quantifying glutamine synthetase activity in the human brain. EJNMMI Res. 2020;10(1):146. 33270177 10.1186/s13550-020-00731-0 PMC7714883

[40] Goldbecker A, Buchert R, Berding G, Bokemeyer M, Lichtinghagen R, Wilke F, et al. Blood–Brain barrier permeability for ammonia in patients with different grades of liver fibrosis is not different from healthy controls. J Cereb Blood Flow Metab. 2010;30(7):1384–93. 20216550 10.1038/jcbfm.2010.22 PMC2949228

[41] Heijtel DFR, Petersen ET, Mutsaerts HJMM, Bakker E, Schober P, Stevens MF, et al. Quantitative agreement between [15O]H2O PET and model free QUASAR MRI-derived cerebral blood flow and arterial blood volume. NMR Biomed. 2016;29(4):519–26. 26876426 10.1002/nbm.3480

[42] van Golen LW, Kuijer JPA, Huisman MC, IJzerman RG, Barkhof F, Diamant M, et al. Quantification of cerebral blood flow in healthy volunteers and type 1 diabetic patients: comparison of MRI arterial spin labeling and [(15)O]H2O positron emission tomography (PET). J Magn Reson Imaging JMRI. 2014;40(6):1300–9. 24214919 10.1002/jmri.24484

[43] Vestergaard MB, Lindberg U, Aachmann-Andersen NJ, Lisbjerg K, Christensen SJ, Rasmussen P, et al. Comparison of global cerebral blood flow measured by phase-contrast mapping MRI with 15O–H_2_O positron emission tomography. J Magn Reson Imaging. 2017;45(3):692–9. 27619317 10.1002/jmri.25442 PMC5324556

