# Supplementary figures and images for: Impact of image reconstruction on cerebral blood flow measured with 15O-water positron emission tomography
Source: EJNMMI Phys. 2025 Jun 6;12:52. doi: 10.1186/s40658-025-00760-5 (PMC12144024; doi:10.1186/s40658-025-00760-5)

# Whole Brain

(A) Regional Quantification

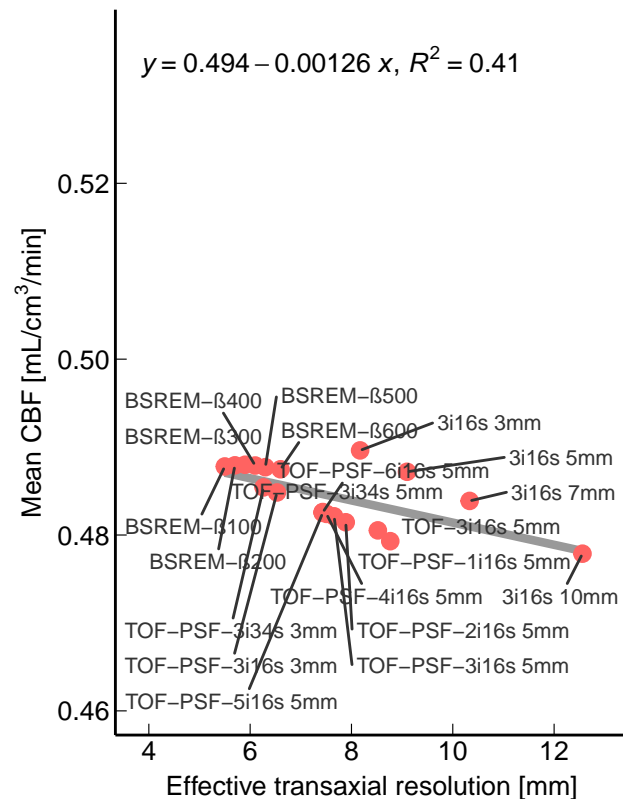

(B) Voxel-wise Quantification

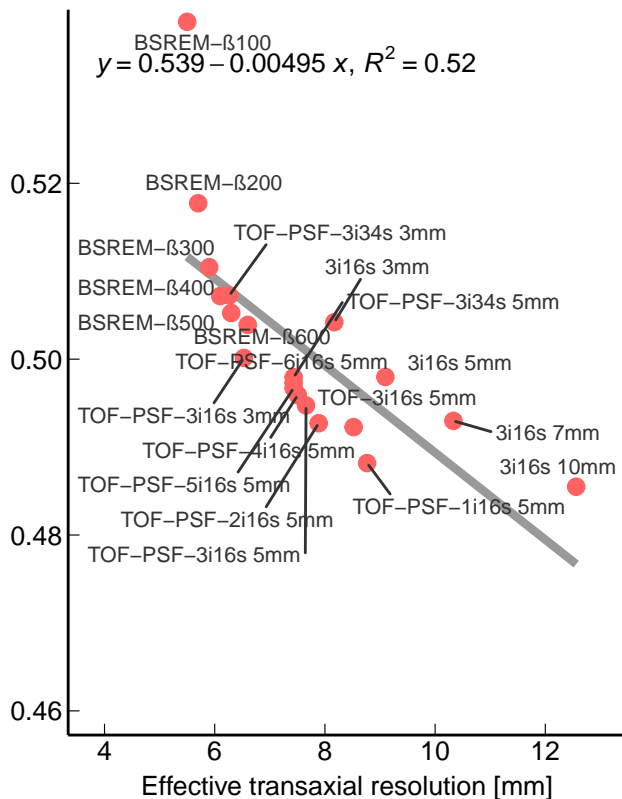

Supplement: Supplementary file 3 — Supplementary Material 3. [file 40658_2025_760_MOESM3_ESM.pdf]
